# Three-Dimensional Modeling of the Structural Microenvironment in Post-Traumatic War Wounds

**DOI:** 10.1007/s13770-021-00355-y

**Published:** 2021-08-07

**Authors:** Gregory T. Christopherson, Jaira F. de Vasconcellos, John C. Dunn, Daniel W. Griffin, Patrick E. Jones, Leon J. Nesti

**Affiliations:** 1 Orthopaedic Research Group, National Institute of Arthritis, Musculoskeletal and Skin Disease, National Institutes of Health, 9000 Rockville Pike, Bethesda, MD 20892 USA; 2 Clinical and Experimental Orthopaedics, Department of Surgery, Uniformed Services University of Health Sciences, 4301 Jones Bridge Road, Bethesda, MD 20814 USA; 3 Henry M. Jackson Foundation for the Advancement of Military Medicine, 6720A Rockledge Drive, Bethesda, MD 20817 USA; 4 Department of Biology, James Madison University, 951 Carrier Drive, Harrisonburg, VA 22807 USA; 5 Department of Orthopaedic Surgery, Walter Reed National Military Medical Center, 104 Wood Rd, Bethesda, MD 20814 USA

**Keywords:** War wounds, Heterotopic ossification, Fibrosis, Stem cells, Trauma

## Abstract

**BACKGROUND:**

The development of post-traumatic heterotopic ossification (HO) is a common, undesirable sequela in patients with high-energy (war-related) extremity injuries. While inflammatory and osteoinductive signaling pathways are known to be involved in the development and progression of post-traumatic HO, features of the structural microenvironment within which the ectopic bone begins to form remain poorly understood. Thus, increasing our knowledge of molecular and structural changes within the healing wound may help elucidate the pathogenesis of post-traumatic HO and aid in the development of specific treatment and/or prevention strategies.

**METHODS:**

In this study, we performed high-resolution microscopy and biochemical analysis of tissues obtained from traumatic war wounds to characterize changes in the structural microenvironment. In addition, using an electrospinning approach, we modeled this microenvironment to reconstitute a three-dimensional type I collagen scaffold with non-woven, randomly oriented nanofibers where we evaluated the performance of primary mesenchymal progenitor cells.

**RESULTS:**

We found that traumatic war wounds are characterized by a disorganized, densely fibrotic collagen I matrix that influences progenitor cells adhesion, proliferation and osteogenic differentiation potential.

**CONCLUSION:**

Altogether, these results suggest that the structural microenvironment present in traumatic war wounds has the potential to contribute to the development of post-traumatic HO. Our findings may support novel treatment strategies directed towards modifying the structural microenvironment after traumatic injury.

**Supplementary Information:**

The online version contains supplementary material available at 10.1007/s13770-021-00355-y.

## Introduction

Following orthopaedic trauma from high-energy mechanisms, such as combat-related blast injuries or terrorist incidents (for example, the 2013 Boston Marathon bombing), impacted tissues become fibrotic and are populated with many different cell types including mesenchymal progenitor cells (MPCs) [1, 2]. We have previously reported the isolation and characterization of MPCs from high-energy (war) traumatized muscle tissues, and demonstrated their ability to tri-differentiate into adipocytes, osteoblasts or chondroblasts [1]. MPCs recruitment to sites of blast injury is thought to be a part of a normal healing process, however these cells may also contribute to the development of post-traumatic heterotopic ossification (HO) [3]. While most research has been focused on identifying key biochemical and chemokine molecules that may influence post-traumatic HO, much less is known about the traumatic wound bed’s structural microenvironment and its potential impact on the MPCs that participate in the healing response.

After penetrating blast trauma, the wound healing process is initiated and results in tissue regeneration and repair [4, 5]. While the regenerative response that creates normal tissue is the most desirable, a predominance of tissue repair (fibrosis) is usually observed [4, 6]. The tissue repair process produces a less functional, but durable tissue with a large fibrotic component [4, 7], which is effective in facilitating wound closure and protecting the body from outside elements, but it possesses little of the normal tissue’s physical, chemical or functional qualities [7, 8]. For the most part, the physical properties are grossly stiffer and less compliant than normal muscle, fat and fascia, however softer and more compliant than bone [4, 7, 8]. Importantly, while fibrosis is thought to be a prelude to the development of post-traumatic HO, the molecular mechanisms underlying the onset of post-traumatic HO remain mostly unknown.

It is known that the physical properties of the surface substrate influence on the adhesion, proliferation and differentiation potential of progenitor cells [9–11]. Therefore, it is possible that MPCs cultured on a substrate that reflect traumatized wound beds, will display properties that are different from the same cell population grown on standard tissue culture plastic. While several components of the extracellular matrix (ECM) are known to cell attachment and growth, type I collagen (COLI) is known to play a predominant role in the wound-healing environment [4, 8]. COLI is present in a highly ordered quaternary structure, however in fibrotic tissues, it has been shown to assume a more random and disorganized orientation [4, 7]. As such, we hypothesized that the fibrotic structural microenvironment is predominantly comprised of COLI nanofibers in post-traumatic war wounds and may have the potential to influence primary MPCs adhesion, proliferation and fate towards the osteogenic lineage. Here we investigated the physical characteristics of the traumatic wound bed, and how those physical properties influenced primary MPCs and may contribute to the development of ectopic bone formation.

## Materials and methods

### Ethics statement and clinical samples

Tissue samples from penetrating traumatic blast extremity injuries sustained by military personal during war conflict were collected approximately 1–2 weeks after injury at the time of surgical debridement at the Walter Reed National Military Medical Center. Uninjured, control muscle samples, were obtained from hamstring harvest of patients undergoing ligamentous knee reconstruction surgery at the Walter Reed National Military Medical Center. The Walter Reed National Military Medical Center Institutional Review Board approved this tissue procurement protocol. The amount of tissue debrided was determined by the operative surgeon. After surgical debridement of the tissue, de-identified samples were placed in a sterile container and transported on ice to the laboratory for processing. All samples were divided for (i) snap freezing in liquid nitrogen, (ii) fixation for scanning electron microscopy evaluation and (iii) isolation of primary mesenchymal progenitor cells (MPCs).

### Isolation of primary mesenchymal progenitor cells (MPCs) and cell viability analysis

MPCs were isolated as previously described [1, 12]. Briefly, fat, fascia, other connective and necrotic areas of tissue were dissected away from the healthy margin of the debrided muscle sample. Part of the tissue (~ 0.5 cm) was washed three times in Hanks’ Balanced Salt Solution (HBSS, Gibco, Carlsbad, CA, USA), minced and incubated with 0.5 mg/mL Collagenase type II (Worthington Biochemical, Lakewood, NJ, USA) for two hours at 37 °C with agitation. Following incubation, the tissue was filtered through 100 μM followed by 40 μM cell strainers (Falcon/Corning, Corning, NY, USA), cells were pelleted by centrifugation and resuspended in growth medium (Dulbecco’s Modified Eagle Medium supplemented with 10% fetal bovine serum (FBS) and 3X Penicillin/Streptomycin and Fungizone, Gibco). The freshly isolated MPCs were plated in 3D collagen nanofibers (collagen scaffolds), 2D collagen (collagen coated) and standard non-coated tissue culture polystyrene (TCPs) dishes for 2 h and then washed with HBSS to remove the non-adherent cells. Subsequently, cells were either (i) expanded for ~ 11–21 days and used in downstream experiments or (ii) submitted for the live/dead fluorescent viability assay for confirmation of viable cells. Briefly, live/dead two-color assay (ThermoFisher Scientific, Waltham, MA, USA) was used to determine viability of cells based on membrane integrity. Freshly isolated MPCs were stained for 45 min at room temperature following manufacture’s recommendations, and subsequently washed 3 times with Phosphate Buffered Saline (PBS). Samples were analyzed under fluorescent microscope for viability using a Zeiss Axio Observer Z1(Carl Zeiss, Thornwood, NY, USA).

### Scanning electron microscopy (SEM)

Surgically debrided tissue was fixed and processed for SEM imaging. First, fresh tissues were sectioned into approximately 5 mm^3^ pieces, fixed in 2.5% paraformaldehyde (PFA)/glutaraldehyde in 0.1 M sodium cacodylate buffer (pH 7.4; Electron Microscopy Sciences, Hatfield, PA) for 2 h at room temperature followed by a second fixation in 1% osmium tetroxide (Electron Microscopy Sciences) for 20 min. Subsequently, samples were rinsed with ddH_2_O and serially dehydrated in a 50/75/80/90/100/100% ethanol series before critical-point drying in hexamethydisilazane (HMDS; Electron Microscopy Sciences). The desiccated samples were coated with gold using a sputter coater (Balzers; Schaumburg, IL, USA) and surface topography was examined by scanning electron microscope (S-4800; Hitachi, Troy, MI, USA, in the Biomedical Engineering and Physical Science Shared Resource, NIBIB, National Institutes of Health, Bethesda, MD, USA) at 5 kV with various magnifications.

### Determination of fiber diameter

Fiber diameter was calculated from a series (10 random fields) of SEM images from 3 independent donors using Image J software (NIH) as previously described [13, 14].

### Creation of nanofiber scaffolds

In order to reconstitute the microenvironment of traumatized tissue, type I bovine collagen (Elastin Products Company, Owensville, MO, USA) was dissolved in 1,1,1,3,3,3-hexafluoro-2-propanol (Sigma Aldrich, St. Louis, MO, USA) and electrospun as previously described [13, 14]. The collagen nanofiber matrix was adhered to coverslips or polymeric culture dish inserts using a medical silicone adhesive (Factor 2, Lakeside, AZ, USA), and briefly crosslinked in 25% glutaraldehyde vapor for 10 min. A 2.5% concentration of collagen electrospun at 0.8 mL/hr yielded a fiber diameter of 100 microns or less and had a 0.89 correlation coefficient with the fibers seen in traumatized muscle. To provide a non-collagen nanofibrous matrix comparison, 1.6 g of poly L-lactic acid (PLLA, Polysciences, Warrington, PA, USA) was dissolved in 10 mL chloroform and 1 mL of DMF followed by vortex-mixing overnight at room temperature and then electrospun at 0.4 mL/hr. When indicated, commercially available 6-well polycaprolactone (PCL) nanofiber plates (700 nm diameter fibers randomly oriented) were also used for the experiments (Nanofiber Solutions, Hilliard, OH, USA).

### Osteogenic differentiation

MPCs were induced to undergo osteogenic differentiation, as previously described [1, 2]. Briefly, multiprogenitor cells were seeded at a density of 5000 cells/cm^2^ and treated for 4-weeks with osteogenic medium, consisting of Dulbecco’s Modified Eagle Medium with 10% FBS supplemented with 10 mM β-Glycerol phosphate (Sigma-Aldrich, St. Louis, MO, USA), 50 μg/mL ascorbic acid (Sigma-Aldrich), 10 nM 1,25-di-hydroxyvitamin D_3_ (BIOMOL International, Plymouth Meeting, PA, USA) and 0.01 μM dexamethasone (Sigma-Aldrich). After induction, monolayered cultured cells were fixed and stained with use of a kit for alkaline phosphatase activity (Sigma-Aldrich) or 2% Alizarin Red S at pH 4.2 (Sigma-Aldrich) for evidence of a mineralized matrix (osteogenic differentiation) as previously described [1, 15].

### Flow cytometry

MPCs derived from adhesion to the different reconstituted surfaces were removed by Accutase (Sigma-Aldrich) treatment and harvested cells were washed, resuspended in PBS supplemented with 0.1% FBS (Life Technologies, Grand Island, NY, USA) containing the following conjugated mouse IgG1, κ anti-human monoclonal antibodies (BD Biosciences, San Jose, CA, USA): CD14-PE, CD73-PE, CD90-PE, CD105-PE (1:100 dilution) for 1 h at 4 °C. After incubation, cell suspensions were washed twice and cell pellet resuspended in 1% paraformaldehyde for analysis by flow cytometry (Fortessa, BD Biosciences). Subsequent analysis was performed using FlowJo software (Tree Star, Ashland, OR, USA).

### MPCs adhesion

Cells were isolated from approximately 200 mg of debrided muscle tissue onto eight 15 cm culture plates coated with 3D collagen nanofibers (n = 3), 2D collagen (n = 3) and tissue culture polystyrene (TCPs; n = 2). Cells adhered for two hours and then washed with PBS three times. After incubation, cells were counted in 10 random fields per plate at 32X magnification. Additionally, a live dead assay was performed on cells grown on 3D collagen nanofibers and 2D collagen coated plates to investigate cell viability.

### MPCs proliferation

BD Horizon Violet Cell Proliferation Dye (VPD450; BD Bioscience, Bedford, MA, USA) was used to assess the proliferative capacity of MPCs following manufacturer’s instructions (BD Bioscience). Briefly, 1 mL of cell suspension was incubated with 1 µM dye for 15 min at 37 °C. After incubation, cells were washed twice by centrifugation resuspending the pellet in PBS. A total number of 100,000 cells were seeded onto either 3D collagen nanofibers (collagen scaffolds), 2D collagen (collagen coated) and tissue culture polystyrene (non-coated standard) dishes and incubated for an additional 7-days prior to harvesting and processing for flow cytometry. Only viable cells give a fluorescent signal at ~ 450 nm wavelength, and the signal intensity decreases approximately 50% during cell division. Fluorescence was analyzed in a BD LSRII flow cytometer (BD Biosciences) at the Flow Cytometry Core, Biomedical Instrumentation Center, Uniformed Services University of the Health Sciences.

### Treatment with TGFß inhibitors

Primary MPCs were seeded at a density of 5000 cells/cm^2^ and treated for 4-days with TGFß [10 ng/mL] (Sigma-Aldrich) and the TGFß inhibitors SB431542 [3 uM], LY2157299 [3 uM], Halofuginone [30 nM] or SIS3 [20 uM] (inhibitors were commercially available from MedChem Express, Monmouth Junction, NJ, USA). DMSO alone [20 uM] was used as vehicle control and TGFß [10 ng/mL] alone was used as experimental control. Experiments were performed in 4 independent donors.

### RNA isolation and quantitative PCR (q-RT-PCR) analysis

Gene-expression analyses for fibrotic (*ACTA2*, *COL1A1* and *FN1*) and the osteogenic marker *CBFA1* were performed following 4-days of treatment with each respective TGFß inhibitor. RNA was extracted using TRIzol (Thermo Fisher Scientific/Invitrogen) following manufacturer’s instructions and purified using RNeasy Mini-columns (Qiagen, Germantown, MD, USA). RNA concentration was measured with a Nanodrop spectrophotometer (ThermoFisher Scientific), where RNA quality corresponded to an A260/280 value of at least 1.8 followed by cDNA synthesis. Relative gene expression analyses were performed by q-RT-PCR with an Applied Biosystems QuantStudio 7 Flex real-time PCR detection system (Applied Biosystems, Foster City, CA, USA). Gene expression was normalized using *GAPDH* as an internal housekeeping control. The list of primers used in this study can be found on Supplemental Table 1.

### Western blot analysis

To decellularize the muscle tissue, an approximately 2 cm^2^ piece of traumatized muscle was placed in 1% Sodium dodecyl sulfate (SDS) on a rotating mixer, with solution changes every 24 h for 3 days. The muscle was then rinsed twice with PBS and placed in a 0.1% Triton X-100 solution overnight to remove residual SDS. Finally, the tissue was rinsed three times (1h each) with PBS to remove any remaining surfactant. Two sets of decellularized traumatized muscle tissue lysates (10 and 5 µg) were separated into 10% precast SDS-PAGE gels (Bio-Rad, Hercules, CA, USA) side by side. Separated proteins from the gel were transferred onto nitrocellulose membrane (Life Technologies). One set of transferred membrane was rinsed with water, fixed with 100% methanol, stained with 0.1% Coomassie Blue 250 in 40% methanol-1% acetic acid, destained with 50% methanol-1% acetic acid, and finally washed with distilled water. The other transferred membrane was incubated using primary type I collagen antibody (Abcam, Cambridge, MA, USA) and secondary alkaline phosphatase (AP)-conjugated anti-mouse antibody (Life Technologies). The antibody specific protein band was visualized by alkaline phosphatase colorimetric development using NBT/BCIP substrates (Bio-Rad).

### Statistical analysis

Replicates are expressed as mean ± standard error values and significance was calculated by one- or two-tailed Student’s t-test as indicated.

## Results

**Fig. 1:**
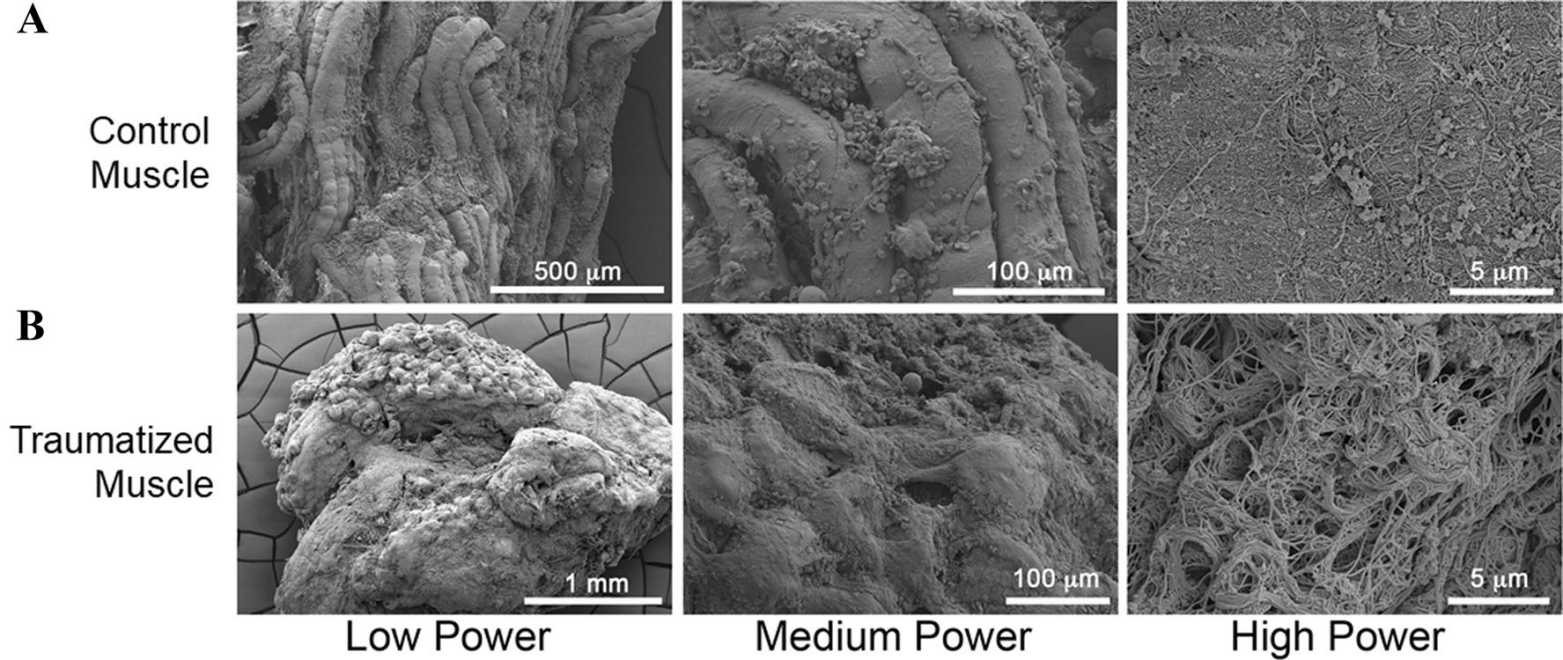
Traumatized muscle displays disarrayed fibers when compared to control muscle. Tissue from either human **A** normal (control) or **B** traumatized muscle tissue was analyzed by scanning electron microscopy. Differing magnifications (Low, Medium, High) show the degree of fiber organization and disorganization in the muscle

To determine the physical characteristics of the structural microenvironment we performed SEM analysis of traumatized muscle compared to uninjured (control) muscle. As shown in Fig. 1, healthy muscle displays a regular fiber array. At the highest magnification normal muscle shows thin, tightly packed fibers. In contrast, traumatized muscle shows a lack of organized fibers at low magnification, with abundant scar matrix at high power, arranged as random nanofibers. At the highest magnification, the disordered ECM structure is clearly evident and distinct in traumatized muscle in contrast to uninjured (control) muscle.

**Fig. 2:**
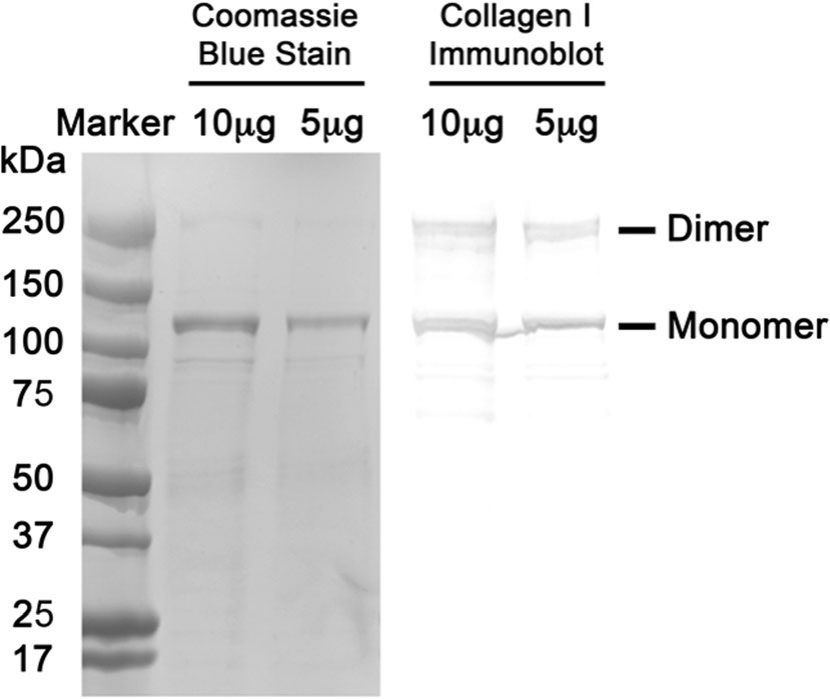
Decellularized traumatized muscle is primarily composed of type I collagen. Traumatized muscle was decellularized and processed for SDS-PAGE. Identical aliquots were electrophoresed side-by-side. The left half of the gel was fixed and stained with coomassie blue while the right half was processed for immunoblotting with type I collagen antibody. Shown is the stained gel aligned with the collagen I immunoblot. The position of the prominent collagen type I band (monomer and dimer) is indicated

To identify the principal components of the human traumatized muscle ECM, primary muscle tissue was decellularized and the principal structural proteins were analyzed by SDS-PAGE. One protein of approximately 110 kDa was most abundant as evident by Coomassie blue staining (Fig. 2, left panel). Since type I collagen is the primary ECM protein secreted by fibroblasts in a reparative scar response [4, 8], we performed immunoblotting with a type I collagen antibody in lanes adjacent to the Coomassie blue stained gel. A prominent band of 110 kDa is detected by the type I collagen antibody, aligning with the Coomassie blue band (Fig. 2, right panel). Thus, our data suggests that the disordered array of nanofibers present in human traumatized tissue is composed of type I collagen, a fibrotic matrix likely created in response to the traumatic injury, and that collagen scaffolds may have value to reconstitute a similar microenvironment, providing an opportunity to investigate the behavior of MPCs in *in vitro* conditions.

**Fig. 3:**
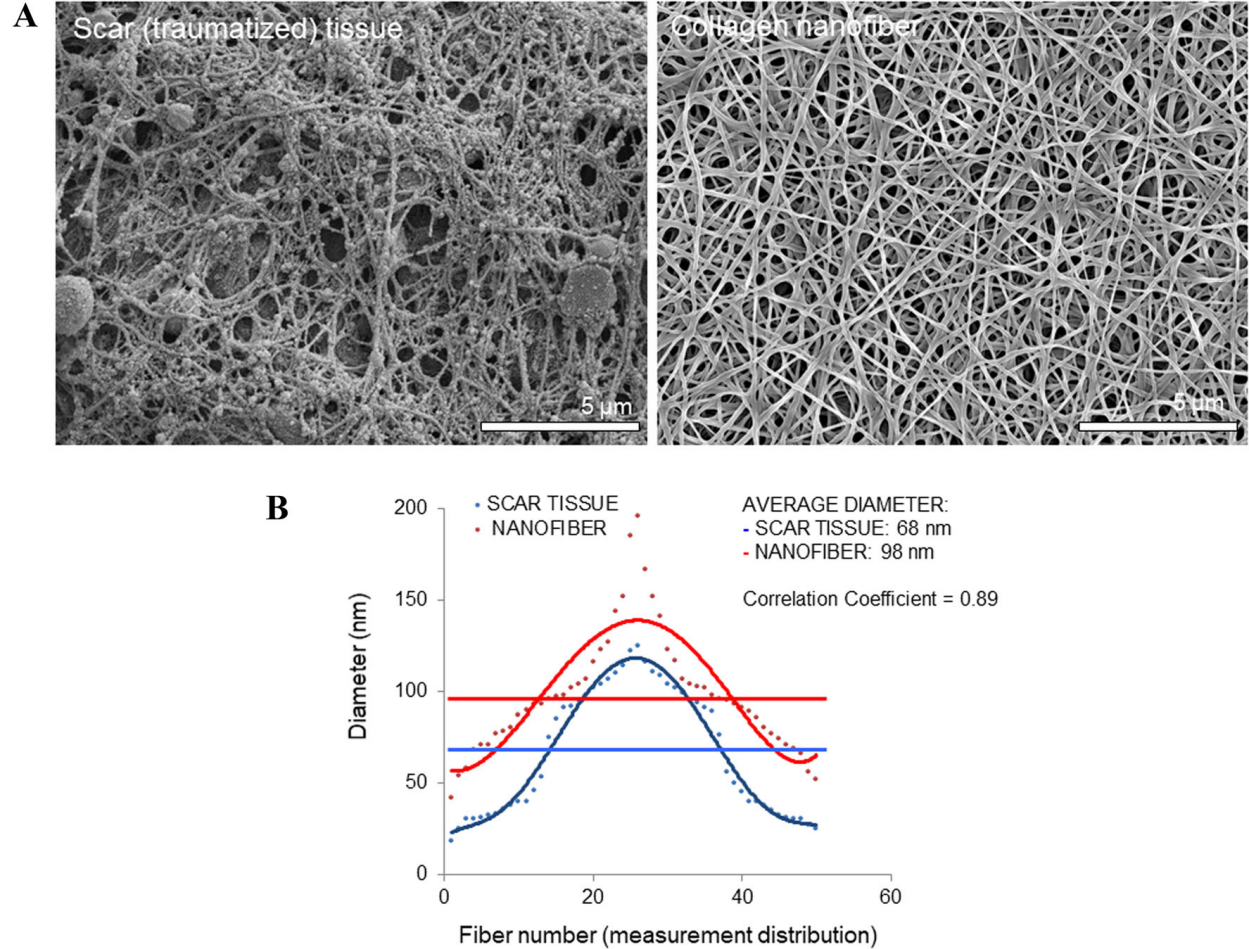
Electrospun type I collagen nanofiber matrix resembles human primary scar/trauma tissue. **A** High magnification scanning electron microscopy of traumatized human skeletal muscle (left) and electrospun collagen I nanofiber (right). **B** Measurements of fiber diameter from traumatized skeletal muscle and electrospun collagen I nanofiber from Panel A images. Measurements are shown as diameter in nanometers (y-axis) *versus* fiber number (x-axis)

Subsequently, we evaluated the physical characteristics of the ECM influencing primary MPCs derived from this human traumatized muscle. To investigate that we electrospun an artificial type I collagen nanofiber scaffold (3D collagen nanofibers). As shown in Fig. 3A, this electrospun type I collagen nanofiber scaffold resembles the disordered ECM in traumatized muscle (scar tissue). The average diameter of the electrospun mesh (3D collagen nanofibers) is similar to the native scar tissue (68 nm for collagen in tissue *vs*. 98 nm for the electrospun collagen, Fig. 3B), with a similar diameter profile. Therefore, this scaffold mimics the microenvironment in traumatized muscle *in vivo*.

**Fig. 4:**
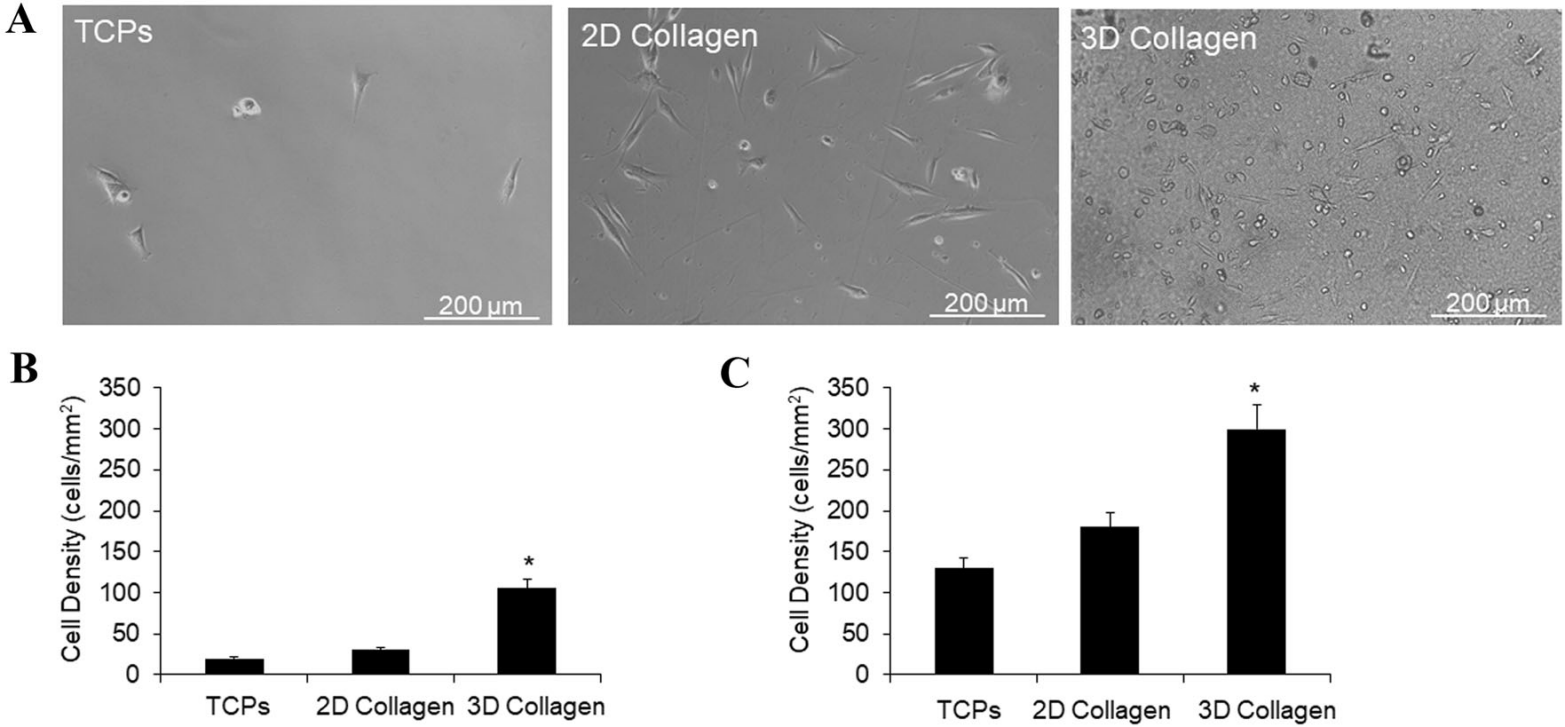
Enhanced adherence and proliferation of MPCs on a type I collagen nanofiber matrix. **A** Representative images and **B** quantification of the number of MPCs isolated following a 2h adherence of digested tissue onto 3 surfaces: tissue culture plastic (TCPs), collagen coated tissue culture plastic (2D collagen) and collagen I nanofiber (3D collagen). Cell numbers were determined by counting the cells in a high power (32X) light microscope field. Ten random fields were counted for each surface. **C** Quantification of the number of MPCs following a 1-week culture on the three surfaces: TCPS, 2D collagen and 3D collagen. Equal numbers of cells were seeded on each surface at time 0. Cell proliferation was assessed using the BD Horizon Violet Cell Proliferation Dye (VPD450; BD Bioscience) following manufacturer’s instructions. Fluorescence was analyzed at the Flow Cytometry Core, Biomedical Instrumentation Center, Uniformed Services University of the Health Sciences. *MPCs* mesenchymal progenitor cells, *TCPs* tissue culture polystyrene, *2D collagen* type I collagen coated TCPs, *3D collagen* type I collagen nanofiber mesh

We next evaluated the adhesion and proliferation profile of primary progenitor cells on the biomimetic 3D collagen nanofibers scaffold. To assess adhesion, crude cellular preparations derived from traumatized muscle were allowed to adhere to the collagen nanofiber matrix. Approximately fivefold more cells adhered to the collagen I nanofiber (3D collagen nanofibers) as compared to collagen coated tissue culture plastic (2D collagen) and tissue culture polystyrene (non-coated standard, TCPs) dishes during our isolation protocol, over a 2h period (Fig. 4A, B). The average cell number per field (mean ± SE) was 30.4 ± 1.14 cells/field on TCPs compared to 31 ± 1.15 cells/field on 2D collagen, and 96 ± 1.96 cells on 3D collagen nanofiber. To test the effects in cell proliferation, an equal number of cells were plated on 3D collagen nanofibers (collagen scaffolds), 2D collagen (collagen coated) and tissue culture polystyrene (non-coated standard) dishes. As shown in Fig. 4C, after 7-days in culture, a higher number of cells was observed on 3D collagen nanofibers (collagen scaffolds) compared to the other tissue culture conditions.

**Fig. 5:**
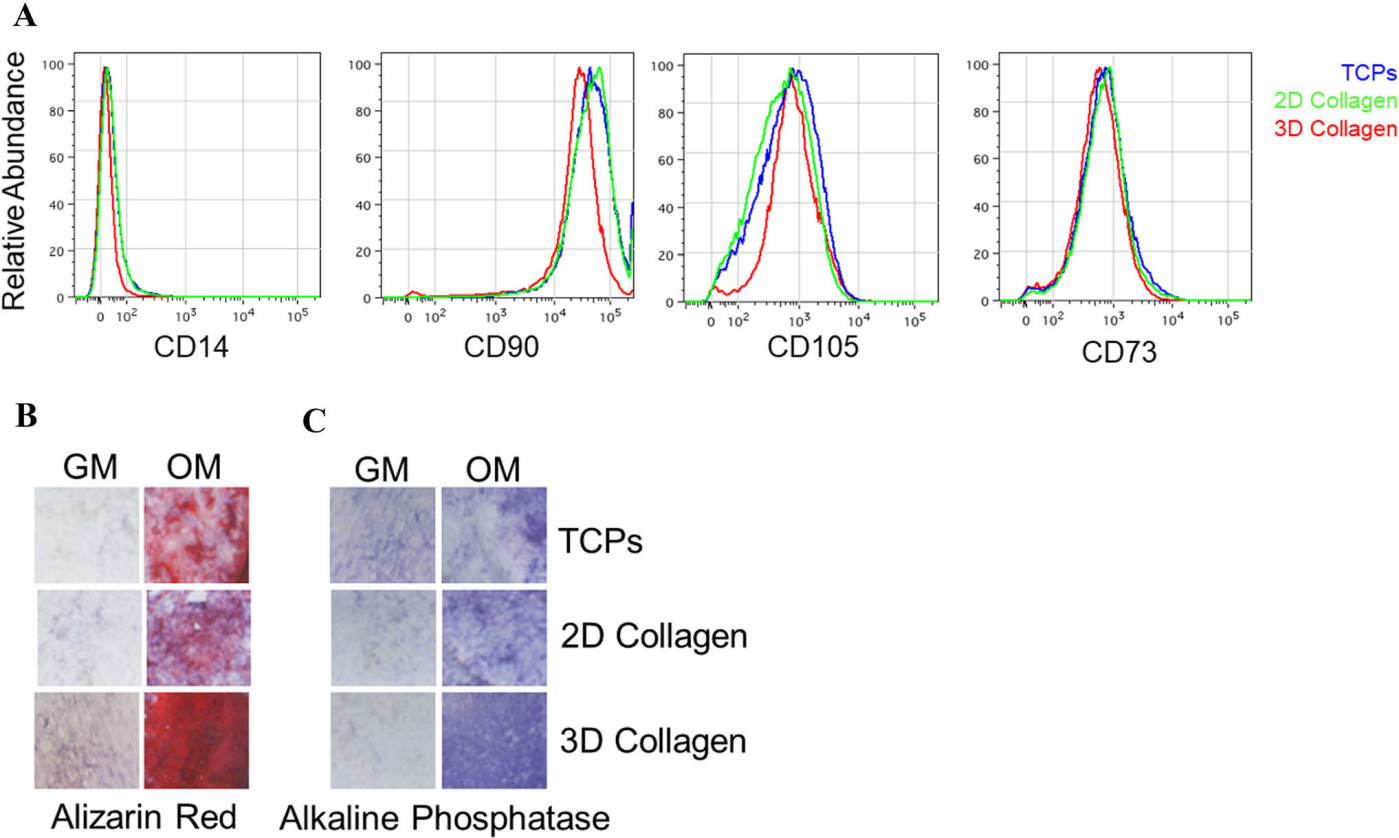
MPCs cultured on type I collagen nanofiber plates displayed similar surface proteins as MPCs cultured on tissue culture polystyrene and demonstrated enhanced osteogenesis differentiation. **A** MPCs were isolated on the three surfaces (TCPS, 2D collagen and 3D collagen). Following a 2h adherence the cells were processed for flow cytometry. The cell-surface markers used were CD105, CD90, CD73 and CD14. Shown are the histograms for each marker antibody (y-axis = cell number, x-axis = fluorescence intensity). **B, C** MPCs isolated on collagen 1 nanofiber and plated on the three surfaces (TCPs, 2D-collagen and 3D collagen) were cultured either in growth media (GM) or osteogenic media (OM). After 3-weeks the cells were fixed and processed for **B** Alizarin red staining for matrix mineralization or **C** alkaline phosphatase expression. *MPCs* mesenchymal progenitor cells, *TCPs* tissue culture polystyrene, *2D collagen* type I collagen coated TCPs, *3D collagen* type I collagen nanofiber mesh

To determine if the cells isolated from traumatized muscle via adherence to a 3D type I collagen nanofiber (collagen scaffolds) were predominately MPCs, the adherent cells were analyzed using flow cytometry. The markers CD73, CD90 and CD105, known to represent a mesenchymal stem cell lineage isolated from muscle [1, 12, 16], were used to compare cells isolated from traumatized muscle following adherence to collagen I nanofiber (3D collagen nanofibers) plates, collagen coated tissue culture plastic (2D collagen) and tissue culture polystyrene (non-coated standard) plates. Cells isolated by adherence to these three surfaces display very similar flow cytometry profiles (Fig. 5A). Additionally, the cells from all three conditions were negative for the hematopoietic marker CD14.

To determine the potential contribution of the structural microenvironment toward the development of ectopic bone or post-traumatic HO, we assessed the osteogenic differentiation capability of MPCs cultured on the biomimetic scaffold (3D collagen nanofibers) in comparison to collagen coated tissue culture plastic (2D collagen) and tissue culture polystyrene plates (non-coated standard, TCPs). MPCs isolated by adherence to type I collagen 3D nanofibers were split and cultured on each of the three surfaces in growth media or osteogenic media for 13 days. The cells were fixed and assessed by histology for mineralization (Alizarin Red staining) and alkaline phosphatase expression. As shown in Fig. 5B and C, mineralization was higher in cells cultured on collagen scaffolds (3D collagen nanofibers) in the presence of osteogenic media compared to cells grown on collagen coated tissue culture plastic (2D collagen) and tissue culture polystyrene (non-coated standard, TCPs), where the level of mineralization was comparable. As expected, osteogenesis was not detected in cells cultured in regular growth media.

Finally, we have previously shown that TGFß levels were elevated in the soft tissues of war-traumatized extremity injuries [17]. Since TGFß mediates most of the initial inflammatory and wound healing response in the traumatized muscle bed and aiming to investigate our findings in a clinically-relevant setting, we examined the effects of addition of TGFß alone or addition of TGFß + TGFß targeted inhibitors on the expression of the fibrotic markers (*ACTA2*, *COL1A1* and *FN1*) and the osteogenic regulator *CBFA1* on human primary MPCs cultured for 4-days in 3-dimensional commercial polycaprolactone (PCL) nanofiber plates. PCL nanofibrous scaffolds have been previously shown to support human mesenchymal stem cells to differentiate into adipogenic, chondrogenic or osteogenic lineages upon culture in each respective differentiation media [18]. We decided to validate our findings using a commercially available PCL nanofiber plate to increase the translational impact and potential for reproducibility of our study. The commercial PCL nanofiber plate used on these experiments have fibers on a random orientation, which as we have shown above (Fig. 3) mimics the fibers formed on the fibrotic/scar tissue following a traumatic injury *in vivo*. In addition, use of the PCL matrix eliminates any contribution of potential bioactivity imparted by type I collagen fibers.

**Fig. 6:**
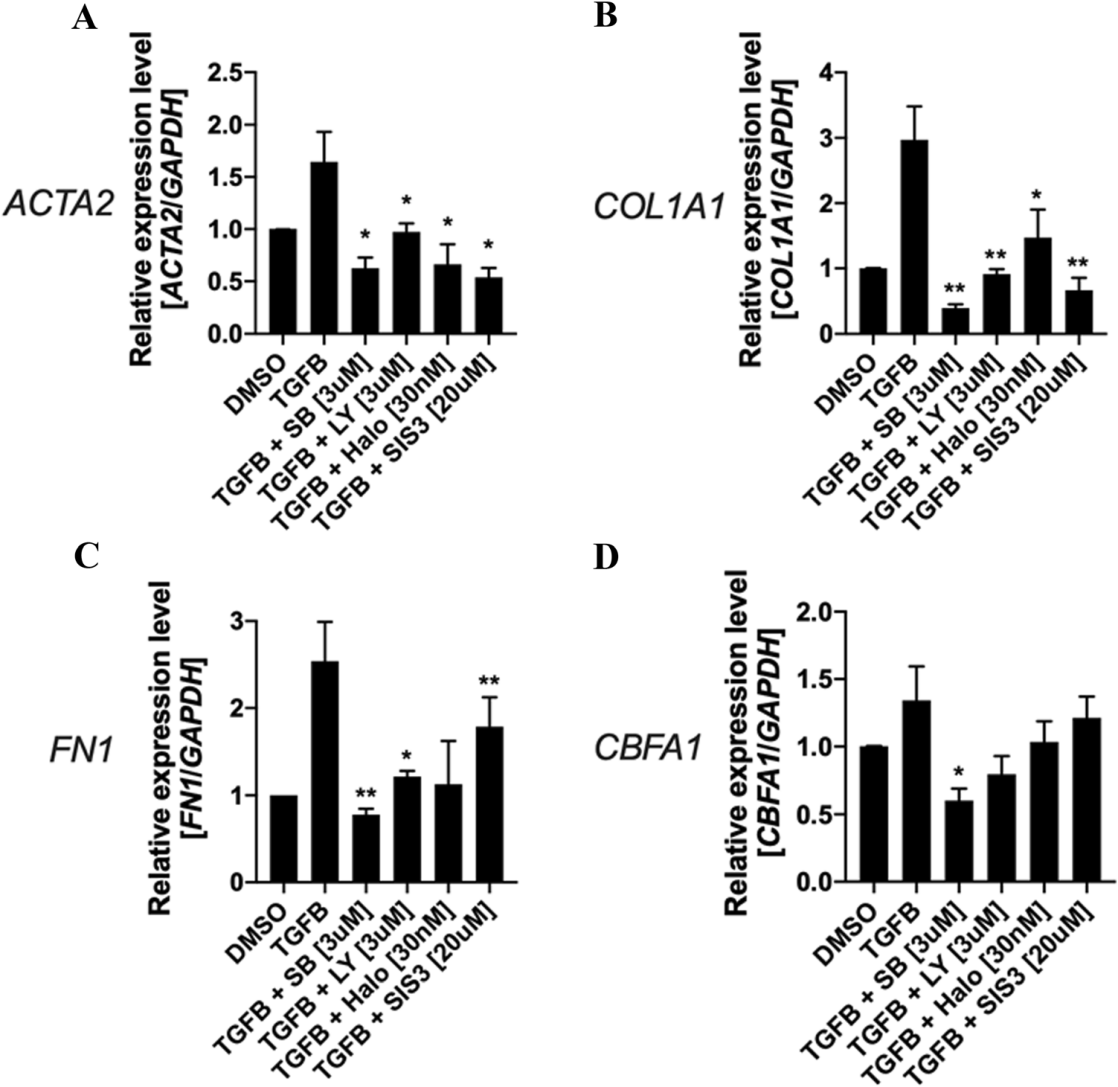
Quantitative PCR (q-RT-PCR) analysis of the fibrotic markers **A** *ACTA2*, **B** *COL1A1* and **C** *FN1*, and **D** the osteogenic marker *CBFA1*. MPCs were cultured on randomly oriented PCL nanofiber plates and treated with TGFß [10 ng/mL] alone (control), TGFß [10 ng/mL] + SB431542 [3 uM], TGFß [10 ng/mL] + LY2157299 [3 uM], TGFß [10 ng/mL] + Halofuginone [30 nM] and TGFß [10 ng/mL] + SIS3 [20 uM] for 4-days followed by RNA extraction, cDNA synthesis and q-RT-PCR analysis. Gene expression was normalized using *GAPDH* as an internal housekeeping control. DMSO alone was used as vehicle control. Results represent the average of 3 independent donors. *p < 0.05, **p ≦ 0.01, T-test 1-tail where treatments were compared to the TGFß treatment alone. *MPCs* mesenchymal progenitor cells, *SB* SB431542, *LY* LY2157299, *Halo* Halofuginone

As shown in Fig. 6, treatment with TGFß [10 ng/mL] alone consistently increased the fibrotic markers (mean ± SD, 1-tail Student’s t-test) *ACTA2* (Control: 1.00 ± 0.00; TGFß treatment: 1.64 ± 0.58; *p* = 0.06), *COL1A1* (Control: 1.00 ± 0.00; TGFß treatment: 2.97 ± 1.01; *p* = 0.02) and *FN1* (Control: 1.00 ± 0.00; TGFß treatment: 2.54 ± 0.91; *p* = 0.02), and the osteogenic regulator *CBFA1* (Control: 1.01 ± 0.01; TGFß treatment: 1.34 ± 0.50; *p* = 0.14) compared to the DMSO alone (vehicle control) treatment*.* Additionally, the addition of TGFß inhibitors consistently down-regulated the expression of *ACTA2* (TGFß treatment: 1.64 ± 0.58; SB431542: 0.63 ± 0.21, *p* = 0.02; LY2157299: 0.97 ± 0.16, *p* = 0.04; Halofuginone: 0.66 ± 0.39, *p* = 0.04; SIS3: 0.54 ± 0.18, *p* = 0.02), *COL1A1* (TGFß treatment: 2.97 ± 1.01; SB431542: 0.39 ± 0.11, *p* = 0.01; LY2157299: 0.91 ± 0.15, *p* = 0.01; Halofuginone: 1.47 ± 0.85, *p* = 0.05; SIS3: 0.67 ± 0.38, *p* = 0.01) and *FN1* (TGFß treatment: 2.54 ± 0.91; SB431542: 0.78 ± 0.13, *p* = 0.01; LY2157299: 1.21 ± 0.13, *p* = 0.02; Halofuginone: 1.13 ± 0.99, *p* = 0.09; SIS3: 1.79 ± 0.68, *p* = 0.01; Fig. 6A, C) as well as *CBFA1* (TGFß treatment: 1.34 ± 0.50; SB431542: 0.60 ± 0.18, *p* = 0.03; LY2157299: 0.80 ± 0.27, *p* = 0.11; Halofuginone: 1.04 ± 0.31, *p* = 0.14; SIS3: 1.22 ± 0.32, *p* = 0.34; Fig. 6D) compared to the TGFß alone (control) treatment.

## Discussion

Musculoskeletal injuries due to military conflicts or civilian blast injuries are known to impact the normal healing process, leading to the formation of exuberant scar tissue that can include ectopic bone formation and fibrosis [19]. While the pathogenesis of post-traumatic ectopic bone formation/heterotopic ossification (HO) remains unclear and an area of active investigation, recent observations suggested that putative mesenchymal progenitor cells (MPCs) populating the site of injury may be influenced by the cellular microenvironment extracellular matrix (ECM) [20]. Here we show that physical features of traumatized muscle tissue from which MPCs are isolated are largely characterized by disorganized fibers in contrast to linear and organized normal tissue control counterparts. Furthermore, since our results indicated that type I collagen was present in decellularized trauma tissues, we chose to use type I collagen to model physiological ECM by electrospinning this biomaterial onto glass/plastic surfaces. Importantly, when comparisons were made between the collagen scaffolds and decellularized traumatized muscle tissue, SEM analysis demonstrated several similar disorganized and tangled fibers with minor differences in thickness. Altogether, these results suggest that disorganized fibers provide a suitable surface for MPCs to attach following injury, creating a cellular microenvironment that favors the formation of post-traumatic HO.

We have previously reported that the isolation of primary MPCs is based on a 2h incubation of the cellular slurry for adequate attachment of multipotential progenitors, while minimizing adherence of non-progenitors [1]. The population of cells attaching to tissue culture plastic as a result of this protocol is relatively small in numbers and require expansion around 1–2 weeks [1]. Here we investigated if the MPCs isolated from traumatized muscle tissue display distinct characteristics upon culture on different electrospun collagen plates (3D and 2D) and commercially available PCL (3D) scaffolds.

Freshly isolated MPCs incubated for 2 h demonstrated an increased adherence of viable cells to the 3D collagen scaffolds when compared to 2D collagen coated plastic, suggesting that a cellular microenvironment consisting, at least in part, of type I collagen ECM provides a surface where MPCs are able to adhere efficiently. In addition to enhanced adherence, our data demonstrated that the scaffolds promoted proliferation and the expression of cell surface markers (CD73, CD90, CD105) previously reported for this type of progenitor cells [1, 15]. Increased cell viability has also been previously reported for MSCs cultured on type I collagen nanofibers, suggesting that a favorable growth and survival *in vitro* microenvironment may be provided by the type I collagen nanofibers [21]. In accordance, human MSCs densities tended to be higher in cells cultured on type I collagen nanofibers compared to poly(l-lactic acid) (PLLA) nanofibers or to cells cultured on cover slips [22]. More recently, MSCs grown on electro-spun silk fibroin scaffolds also demonstrated enhanced attachment and proliferation resulting in greater osteogenic potential [23]. Altogether, these results indicate that 3D nanofibers scaffolds support intrinsic properties of human progenitor cells’ (MSCs and MPCs) growth and survival *in vitro*.

In accordance with previously published studies [21, 22], the multipotency of MPCs that was tested by inducing osteogenesis and the ECM microenvironment consisting of type I collagen (3D collagen scaffolds) was observed to enhance the potential of MPCs to undergo osteogenic differentiation compared to 2D collagen coated plastic or non-coated tissue culture plastic. Unexpectedly, we observed that alkaline phosphatase expression was slightly higher on non-coated tissue culture plastic compare to 2D collagen coated plastic or 3D collagen scaffold in general media conditions. The biological significance of this finding still needs to be further investigated. Of note, other factors have been shown to support osteogenic differentiation of MSCs; MSCs cultured in bone morphogenetic protein-2 (BMP-2)-derived peptides immobilized onto nano-hydroxyapatite/PLLA/gelatin 3D nanofibrous scaffolds demonstrated the ability to differentiate into the osteogenic pathway *in vitro* [24]. In addition, TGFß has been shown to play a role in osteogenic differentiation, bone regeneration and formation of HO *in vitro* and *in vivo* [25–27]. Here we showed that fibrotic markers activated (up-regulated) by treatment with TGFß were maintained and, in some cases (*COL1A1* and *FN1*) increased, by culture of primary MPCs on a 3D scaffold. In addition, two different classes of TGFß inhibitors were used in this study: (i) ALK5/TGFß type I receptor inhibitors that target the TGFß receptor by inhibiting both SMAD2 and SMAD3 phosphorylation [SB431542 and Galunisertib/LY2157299] and (ii) SMAD3 inhibitors that specifically target and inhibit SMAD3 phosphorylation [Halofuginone and SIS3]. Since the mechanism of action from these 2 classes of TGFß inhibitors are different, we chose to test 2 inhibitors from each class in order to intrinsically corroborate the results obtained with each individual inhibitor. Remarkably, most ALK5 and SMAD3 inhibitors significantly down-regulated the expression of fibrotic markers, while only SB431542 (ALK5 inhibitor) significantly down-regulated the expression of the osteogenic regulator *CBFA1*. Nevertheless, a trend towards down-regulation of *CBFA1* was observed with all other inhibitors.

While our data demonstrate the potential use of collagen scaffolds for regenerative medicine applications, in particular to better understand the pathogenesis behind the onset of fibrosis following traumatic injuries, some limitations to this study exist. Electrospun collagen was done in a disorganized manner to broadly mimic the ECM of trauma injured tissues. In addition, it has been shown that aligned and non-aligned fibrous scaffolds induced MSCs to differentiate into different lineages [11]. MSCs grown on disorganized fiber scaffolds displayed enhanced osteogenic differentiation compared with cells cultured on aligned fiber scaffolds, suggesting a strong influence of topographical surfaces for divergent differentiation of progenitor cells [11]. Nonetheless, it has also been demonstrated that scaffolds consisting of aligned fibers with incorporated type I collagen enhanced osteogenic potential of rat adipose-derived MSCs [10]. Finally, human MSCs proliferation and osteogenic differentiation abilities were significantly reduced upon prolonged culture, which was significantly recovered by culture in poly L-lactic acid electrospun nanofibrous scaffolds compared to standard culture polystyrene plates [28].

In summary, we have provided novel insights into the physical microenvironment of traumatized musculoskeletal tissue and demonstrated how a biomimetic replicate of this physical environment may influence MPCs growth, proliferation and fate towards the osteogenic lineage. While the use of electrospun 3D nanofiber scaffolds for isolation and growth of mesenchymal cells has been demonstrated for over a decade and is a hallmark of tissue engineering [29–32], we have now demonstrated that a randomly oriented, nanofiber collagen type I scaffold, as a biomimetic, could allow for greater progenitor cell adhesion, increased progenitor cell proliferation and improved progenitor cell differentiation towards an osteoblastic phenotype.

## Supplementary Information

Below is the link to the electronic supplementary material.

Supplementary file1 (DOCX 12 kb)
