# Supplementary material for: Three-Dimensional Modeling of the Structural Microenvironment in Post-Traumatic War Wounds
Source: Tissue Eng Regen Med. 2021 Aug 7;18(6):963–73. doi: 10.1007/s13770-021-00355-y (PMC8599535; doi:10.1007/s13770-021-00355-y)
Supplement: Supplementary file 1 — Supplementary file1 (DOCX 12 kb) [file 13770_2021_355_MOESM1_ESM.docx]

**Supplemental Table 1 –** Primer sequences (5’ to 3’) used for relative gene-expression analysis by quantitative Reverse Transcription Polymerase Chain Reaction (q-RT-PCR).

| **Gene** | **Primer Sequence (5’ to 3’)** |
| --- | --- |
| *ACTA2*-Forward | TCAAGATCATTGCCCCTCCG |
| *ACTA2*-Reverse | CCCGGCTTCATCGTATTCCT |
| *COL1A1*-Forward | GTACATCAGCCCAAACCCCA |
| *COL1A1*-Reverse | TCGCTTCCATACTCGAACTGG |
| *FN1*-Forward | TGACAACTGCCGTAGACCTGG |
| *FN1-*Reverse | TACTGGTTGTAGGTGTGGCCG |
| *CBFA1*-Forward | CCGAGCTACGAAATGCCTCT |
| *CBFA1*-Reverse | TGAAACTCTTGCCTCGTCCG |
| *GAPDH*-Forward | ACAGTCCATGCCATCACTGC |
| *GAPDH*-Reverse | GCCTGCTTCACCACCTTCTT |
